# Fabrications of *L*-Band LiNbO_3_-Based SAW Resonators for Aerospace Applications

**DOI:** 10.3390/mi10060349

**Published:** 2019-05-28

**Authors:** Baofa Hu, Shaoda Zhang, Hong Zhang, Wenlong Lv, Chunquan Zhang, Xueqin Lv, Haisheng San

**Affiliations:** 1 Pen-Tung Sah Institute of Micro-Nano Science and Technology, Xiamen University, Xiamen 361005, China; hubaofa@stu.xmu.edu.cn (B.H.); zhangsd@stu.xmu.edu.cn (S.Z.); zuluhong@foxmail.com (H.Z.); lwl1980@xmu.edu.cn (W.L.); cqzhang@xmu.edu.cn (C.Z.); xqlv@xmu.edu.cn (X.L.); 2 Shenzhen Research Institute of Xiamen University, Shenzhen 518000, China; 3 Intelligent Acoustics (Xiamen) Science and Technology Co., Ltd., Xiamen 361000, China

**Keywords:** surface acoustic wave (SAW), resonator, electron beam lithography, SAW sensing

## Abstract

High frequency surface acoustic wave (SAW) technology offers many opportunities for aerospace applications in passive wireless sensing and communication. This paper presents the design, simulation, fabrication, and test of an *L*-band SAW resonator based on 128° Y-X LiNbO_3_ substrate. The design parameters of SAW resonator were optimized by the finite element (FEM) method and the coupling-of-mode (COM) theory. Electron-beam lithography (EBL) technology was used to fabricate the submicron-scale of interdigital transducers (IDTs) and grating reflectors. The effects of some key EBL processes (e.g., the use of electron beam resist, the choice of metal deposition methods, the charge-accumulation effect, and the proximity-effect) on the fabrication precision of SAW devices were discussed. Experimentally, the LiNbO_3_-based SAW resonators fabricated using improved EBL technology exhibits a Rayleigh wave resonance peaks at 1.55 GHz with return loss about −12 dB, and quality factor *Q* is 517. Based on this SAW resonator, the temperature and strain sensing tests were performed, respectively. The experimental results exhibit a well linear dependence of temperature/strain on frequency-shift, with a temperature sensitivity of 125.4 kHz/°C and a strain sensitivity of −831 Hz/με, respectively.

## 1. Introduction

A surface acoustic wave (SAW) is an acoustic wave traveling along the surface of an elastic material. It is generated by interdigital transducers (IDTs), which are periodic metallic bars, deposited on a piezoelectric material. Lord Rayleigh first described and explained the surface mode of acoustic wave propagation in a piezoelectric material [1]. The main property of the Rayleigh type of SAW is that the great majority of energy is localized in the surface region in a penetration depth less than one or two wavelengths, this means that any external surface perturbation will affect the propagation of SAW and lead to a sensitive change in the frequency response of SAW devices. By means of this sensing mechanisms, SAW devices can be utilized for measurement of surrounding environment parameters, e.g., temperature, pressure, humidity, light intensity, gas composition, and mechanical deformation [2,3,4,5,6,7]. 

**Figure 1:**
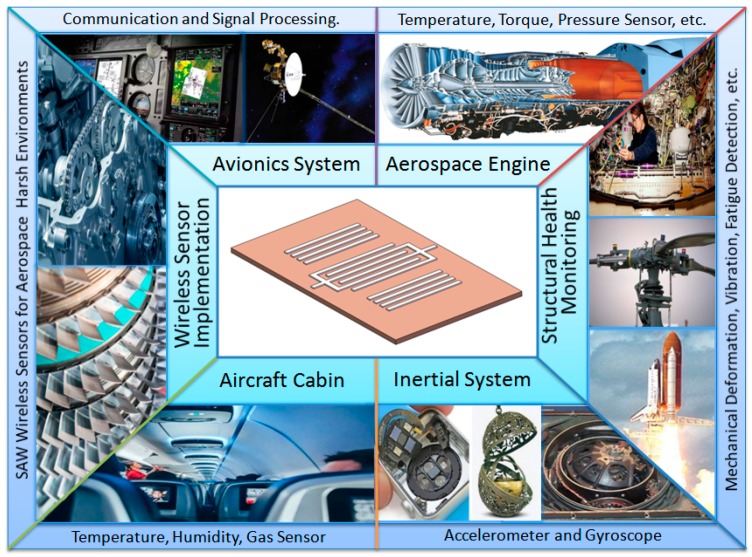
Potential aerospace applications of surface acoustic wave (SAW) sensors.

In comparison with other potentially competitive technologies, SAW technology has special advantages for aerospace applications, as shown in Figure 1. SAW devices can be small, rugged, passive, wireless, and radiation hard and operate with variable frequency and bandwidth [8], which can well meet the aerospace requirements for small, lightweight, inexpensive, and wireless sensors. For example, structural health monitoring (SHM) of aerospace vehicles are greatly important for the safety of the crew and the vehicle. However, some battery-powered sensors are constrained to use in the harsh environment (e.g., extreme temperature and pressure) and the narrow internal space with limited access. One solution to these constraints is to use wireless instead of wired sensors for SHM applications. In contrast to current wireless systems, the small, passive wireless SAW sensors can operate without batteries across a large temperature range to sense physical and chemical parameters [9,10]. Furthermore, multiple sensing parameters also can be obtained using distributed SAW sensor arrays to encode the sensor information and transmission to the receiver [8].

Furthermore, SAW technology can be used to fabricate the resonators and filters in an avionics system [11]. With the development of aerospace communication technology, the demands for high frequency SAW devices are significantly increasing for radio frequency electronic systems, e.g., filters and resonators used in mobile communication systems in the *L*-band (1–2 GHz). According to Sauerbrey function, the frequency shift of SAW devices are proportional to the square of the resonant frequency [2,12]; thus, the high resonant frequency can greatly increase the sensitivity of sensors and reduce the size of devices. 

Generally, there are two ways to increase the resonant frequency of SAW devices. One is to decrease the IDT-bars linewidth and periodic spacing to submicron-scale using high precision lithographic technology (e.g., ultraviolet or electron-beam lithography (EBL) technology). Another is to use piezoelectric substrate with high acoustic velocity. For example, R.-M. et al. have fabricated a 14 GHz AlN/diamond-based SAW resonator with 800 nm of IDT periodic spacing using EBL technology [13]. Hisashi Hatakeyama et al. have fabricated a 5 GHz SAW resonator with 720nm of IDT periodic spacing using 42° Y-X LiTaO_3_ substrate and EBL technology [14]. Although extreme ultraviolet lithography can be used to fabricate deep submicron patterns, the lithography instruments are expensive and mask fabrications are in a high cost. EBL technology is a maskless lithography technique, which can be used to fabricate the micro-nano devices through the direct-writing to define the designed pattern and can achieve the critical dimensions of pattern in less than 100 nm on the polymer. Especially, some commercial EBL systems have been used in connection with scanning electron microscope (SEM) as a more time and cost-effective instrument in lab researches for a flexible study requirement and a low-cost of device fabrication. 

High acoustic velocity of single-crystal piezoelectric materials, i.e., α-quartz, LiNiO_3_, and LiTaO_3_, etc., generally have acoustic velocity in 3000–4000 m/s in favor of the fabrication of GHz SAW devices. It has been reported that the preferred orientation of AlN thin films deposited on diamond or sapphire by reactive magnetron sputtering or metal organic chemical vapor deposition (MOCVD) can achieve the acoustic velocity larger than 10,000 m/s. [15,16,17]. However, these materials have either high fabrication cost or low electromechanical coupling coefficient (EMCC). 

In our work, an *L*-band single-port SAW resonator with resonant frequency of about 1.5 GHz was designed and fabricated. There are two outstanding achievements that show the difference of this work in comparison with previous works. On the one hand, a two-dimensional finite element model (FEM) based on COMSOL Multiphysics^TM^ (COMSOL Inc., Los Altos, CA, USA)and a numerical model based on coupling-of-mode (COM) theory were built to optimize the design of SAW resonator with 128° Y-X LiNbO_3_ as substrate. Some key parameters, such as wave velocity, reflection coefficient, static capacitance, etc., were extracted by FEM analysis. Furthermore, through COM theory the extracted parameters were used to calculate the response properties of devices and finally determine the optimal structure parameters of the device. On the other hand, the electron-beam lithography (EBL) technology was used to fabricate the 1.5 GHz SAW resonators with submicron linewidth (600 nm) by using improved processes. Although the EBL technology has been introduced in some relevant references [18,19,20,21,22], there are few reports on an overall and comparative result that can exhibit experimentally the influences of electron beam resist properties, metal deposition processes, charge-accumulation effect, and proximity-effect on the EBL fabrication precision.

## 2. Design and Simulation Single-Port SAW Resonator

**Figure 2:**
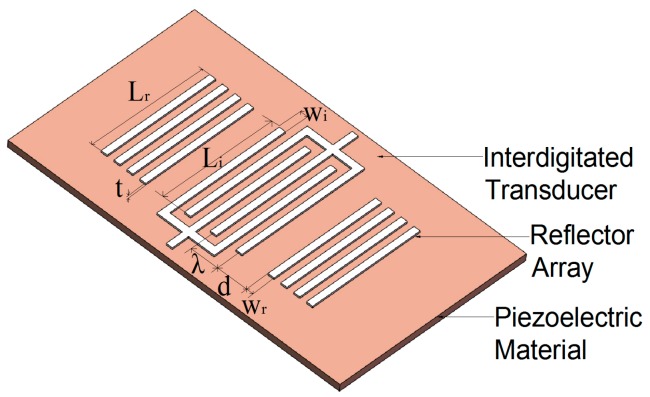
Schematic diagram of SAW resonator and the design parameters of SAW resonator

Figure 2 shows the schematic three-dimensional (3-D) structure of the designed single-port SAW resonator, which consists of a piezoelectric substrate (128° Y-X LiNbO_3_), an Au IDT electrode fabricated on the piezoelectric substrate, and a pair of grating reflectors located on both sides of the IDT [23]. The excited resonant wavelength *λ* of SAW resonator is determined by IDT period spacing *P_i_* and acoustic velocity *ν*. The resonant frequency *f* of the SAW resonator can be expressed as:

(1) $$f\;=\frac{\nu }{\lambda }=\frac{\nu }{P_{i}}$$

The Rayleigh SAWs excited on a 128° Y-X LiNbO_3_ piezoelectric substrate by IDT electrode propagate along the surface and length directions of piezoelectric substrate in two opposite directions. The grating reflectors on both sides of IDT like as two plane mirrors of Fabry-Perot resonant cavity, resulting in the superimposition of two reflected waves each other to form stable and enhanced resonant peaks [24].

**Figure 3:**
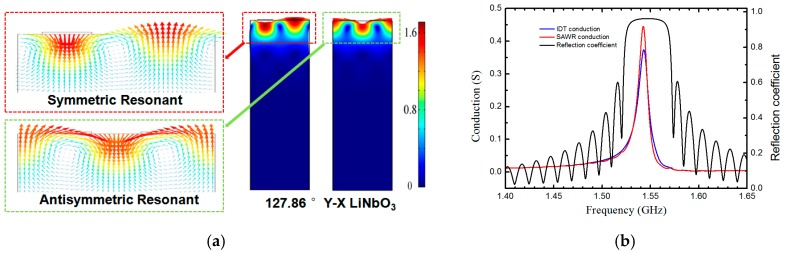
Finite element (FEM) simulations of resonant mode and displacement distribution mapping of SAW (color bar = total displacement (nm)) (**a**), COM simulations of conductance response of IDT and SAW resonator and reflection spectrum of reflectors (**b**).

The optimal structure and size parameters of SAW resonator can be determined by utilizing the finite element (FEM) model and the coupling-of-mode (COM) theory [25,26,27]. Figure 3 shows the FEM simulation of resonant mode and displacement distribution mapping of SAW induced on the 128° Y-X LiNbO_3_ by using COMSOL Multiphysics^TM^ software. The COMSOL simulation uses the triangle meshed elements. In order to reduce the calculation load, the plane strain hypothesis was used to build a simplified two-dimensional model with width in 1*λ* scale and thickness in 5*λ* scale. Based on a two-dimensional model, the period boundary conditions were applied to the right and left boundaries with a width of 1*λ*, and a free surface boundary condition and a Dirichlet boundary condition were applied respectively to surface and lower boundary with a thickness of 5*λ*. It can be seen from Figure 3 that the displacement distribution of Rayleigh wave exhibit symmetric resonant and anti-symmetric resonant characteristics, respectively. At the same time, the amplitude of SAWs decrease rapidly with the increase of depth, and its energy are concentrated in the depth of one wavelength, accounting for the reason why SAWs are very sensitive to external disturbances. Furthermore, the aperture of IDT and length of grating reflectors, the number of bars, the distance between the IDT and the grating reflectors were analyzed by COM theory to investigate their influences on the output signal of device. Meanwhile, two-order effect in high frequency SAW and its influence on the SAW propagation characteristics were also considered. 

**Figure 4:**
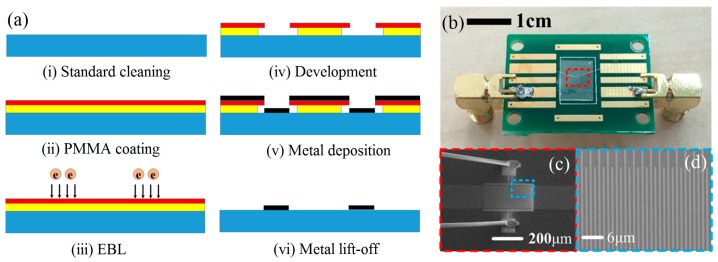
Fabrication process flow of the SAW resonator (**a**); photo of the SAW resonator packaged in printed circuit board (**b**) and scanning electron microscope (SEM) images of the SAW resonator (**c**) and the enlarged IDT structure (**d**).

By the numerical calculation based on COM theory, the parameters of the IDT and the grating reflectors were optimized (e.g., the length of IDT aperture *L_i_*, the pairs of IDT/reflector *N_i_* and *N_r_*, metallization of IDT/reflector *η*, Au thickness *t*) to realize a 1.5 GHz SAW. Table 1 present the optimal design parameters of SAW resonator based on 128° Y-X LiNbO_3_ substrate. The bar-width and pitch-width of the IDT and the grating reflector were determined to be 600 nm, corresponding to a 2.4 μm of period of IDTs. There are 148.5 pairs of input and output IDT and 100 pairs of grating reflectors on each side, corresponding to a length of IDT aperture of 60*λ*. A 60 nm thick Au was designed to fabricate IDT and reflectors. The distance between IDT and reflector was adjusted to 2.4 μm so that the peak of IDT conductance lies in the center of stop-band of reflectors. As a result, a maximum conductance of SAW and thus the optimal energy efficiency were obtained in the resonant structure. The simulated results are shown in Figure 4. It can be seen that the optimal conductance peak of SAW resonator is well located at the stop-band of reflecting gate, and a resonant frequency of 1.52 GHz is determined to match a 2.4 μm of period of IDTs and an acoustic wave speed of around 3700 m/s. Table 2 presents the COM parameters used for the simulation and optimization of the resonator, which were extracted from the FEM analysis [25,26,28,29].

## 3. Fabrication and Testing of SAW Resonator

The Figure 4a shows the fabrication process flow of the SAW resonator. A 4 inch of 128° Y-X LiNbO_3_ wafer (NanoLN, Jinan Jingzheng Electronics Co., Ltd., Jinan, China) was cut into several rectangular substrates with 10 mm (length) × 7 mm (width), and then, the substrates were cleaned in the ultrasonic bath with acetone, ethanol, and deionized water to remove adsorbed dust and surface contamination. Next, an Ar plasma bombardment was used to activate substrate surface and enhance the adhesion of electron beam resist to LiNbO_3_ substrate. For fabricating high-precision of IDT structure, two models of electron beam resists (600 K polymethyl methacrylate (PMMA) and 950 K PMMA, Germany ALLRESIST Co., Ltd., Strausberg, Germany) were used to fabricate the mask pattern. The effects of electron beam resists on the fabrication precision of metal bars will be discussed in subsequent part. The 600 K electron beam resist was firstly spin-coated on LiNbO_3_ substrate with speed of 2000 r/min and then was baked on 150 °C on hot plate for 3 minutes. Following above steps and using same steps to process 950 K electron beam resist on previous substrate. It was measured that the layer thickness of 600 K and 950 K electron beam resists were 240 nm and 120 nm, respectively. Due to the poor conductivity of PMMA resist and LiNbO_3_ substrate, a conductive layer of 8~10 nm Au was sputtered on resist sample to avoid charge accumulation effect before the electron beam exposure, and the extra Au conductive layer should be removed by using KI solution before development. Next, the electron beam writer directly wrote to define the IDTs according to the designed pattern as shown in Figure 2. In this process, the accelerated voltage of electron beam writer was set at 10 kV, and the current was controlled at 50 pA. After EBL process, the resist mask was developed in methyl isobutyl ketone (MIBK)/isopropyl alcohol (IPA) (1:3) solution for 40s and rinsed in IPA for 30 s. Next, Au (60 nm) was deposited on the mask pattern using e-beam thermal evaporation with Cr (5 nm) as adhesive layer using sputtering method. Finally, by using Au/Cr lift-off process, the IDTs and grating reflectors were achieved. For measuring the devices, the LiNbO_3_ substrate with SAW resonators was adhered to a printed circuit board with RF coplanar waveguide connected electrically to the SMA connector. The IDT electrodes of SAW resonator were connected with RF coplanar waveguide circuit through Au wire-bonding as shown in Figure 4b. Figure 4b shows also the SEM image of the complete SAW resonator and the enlarged IDTs structure, respectively. 

The surface morphologies of SAW devices were observed by a Carl Zeiss’s Supra 55 scanning electron microscope (SEM) with a Raith EBL system. The frequency spectrum of the SAW resonator was monitored by a Rohde & Schwarz ZNB8 network analyzer, and the temperature and strain responses of the SAW resonator were measured through the network analyzer interfaced to a computer to record the measured data by a LabVIEW resonance frequency tracking software. 

## 4. Results and Discussion

### 4.1. Effect of Electron Beam Resist on Fabrication Quality

**Figure 5:**
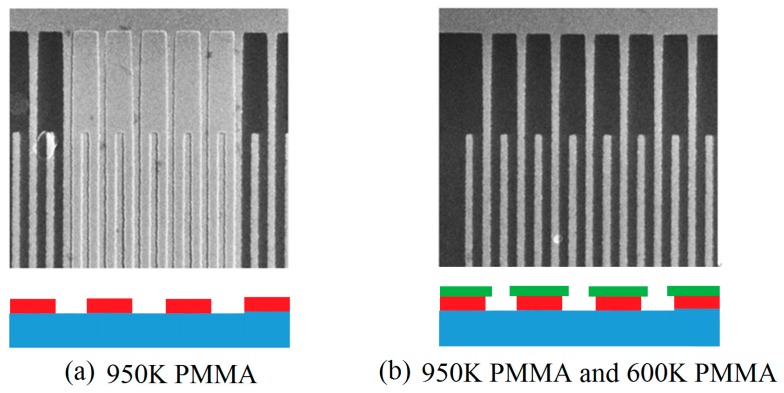
A comparison of the experimental results of metal IDT fabrication using single-layer (**a**) and double-layer resist (**b**).

Generally, the thickness of the resist should be more than two times of that of deposited metal film in order to perform the next lift-off process. If the metal film is deposited by the magnetron sputtering technology, the thickness could be larger because of step coverage; otherwise, the lift-off will be difficult. When the thickness of deposited metal film is greater than 50nm, the exfoliation can be improved by using double-layers resist to obtain the inverted T-shape structure after exposure and development. The relative molecular weight of PMMA electron beam resists has a significant effect on the sensitivity and resolution of exposure. e.g., 600 K PMMA has high sensitivity but low resolution while 950 K PMMA has the reverse properties when compared to 600 K PMMA. Therefore, the cracking size of 600 K PMMA will be larger than that of 950 K PMMA when an electron beam spot is incident on the surface of resist. As a result, the double-layers resist (upper/lower layer = 950 K/600 K PMMA, 950 K is 120 nm and 600 K is 240 nm.) structure will form an inverted T-shape structure after development, which is beneficial to metal lift-off process. Figure 5 shows a comparison of experimental results of metal IDT fabrication using single-layer and double-layers resist. It can be seen that the use of double-layers resist can make a more complete IDT structure than use of single-layer resist.

### 4.2. Effect of Metal Film Deposition Processes on Metal Lift-Off

Vacuum thermal evaporation and RF magnetron sputtering are the common process to deposit metal thin films. Thermal evaporation method is able to create a well directional deposition and poor step coverage, which is beneficial to lift-off and trench filling. In contrast, the sputtering method gives rise to a non-directional deposition good for sidewall and step coverage. 

**Figure 6:**
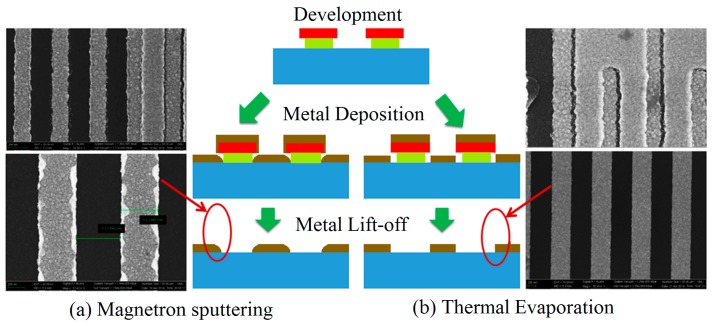
A comparison of the experimental results and of metal IDT fabrication using magnetron sputtering (**a**) and thermal evaporation (**b**).

In the fabrication of IDTs of SAW devices, a double-layer resist was used to form the inverted T-shape structure in order to facilitate the lift-off process. However, a perfect fabrication of IDTs still needs a matched deposition method of metal film. Figure 6 shows a comparison of metal thin films deposited in mask with inverted T-shape structure by magnetron sputtering and thermal evaporation. It can be seen that the thermal evaporation can be used to fabricate a perfect IDT structure after lift-off process, in contrast the sputtering bring about some incomplete bars of IDT with some edge-defects and the residual metal film. A schematic illustration is shown in Figure 6, the sidewall and bottom space in the inverted T-shape structure were covered and filled partly by metal films due to the non-directional deposition of metal atoms in sputtering method, resulting in an incomplete metal exfoliation. 

### 4.3. Effect of Charge Accumulation on Exposure Precision

**Figure 7:**
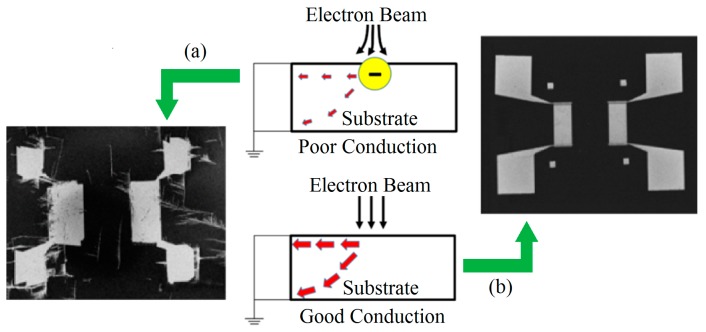
A comparison of developed patterns of e-beam exposure on LiNbO_3_ substrate with (**a**) and without (**b**) Au layer on resist.

When an electron beam resist coated substrate is irradiated by a high beam-current density of electron beam, the charges will accumulates on the surface if the resist or substrate is of insulating material. The accumulated charges will give rise to the deflection of E-beam caused by the electric field produced by the trapped charges, resulting in a considerable amount of pattern displacement and distortion. Generally, piezoelectric materials (e.g., LiNbO_3_, quartz, AlN) are good dielectric materials with poor conductivity. The effect of charge accumulation on exposure accuracy can be reduced by using low accelerated voltage. However, the low accelerated voltage will lead to a large proximity effect, making the exposure precision worse. For a shallow structure, an optimal accelerated voltage (e.g., 10 kV in this work) can be used to achieve a good balance between charge accumulation and proximity effect. For a deep structure, the feasible approach reducing charge accumulation is to deposit a very thin layer of high conductive layer (e.g., Au, Al, Cr) on the surface of the resist in order to dissipate the charge during EBL exposures. It is important to note that a thick metal layer will scatter electrons and limits the fabrication at deep structure. The experiments demonstrate that a 10 nm thick Au layer deposited by sputtering can alleviate the charge accumulation effect obviously without affecting the resolution of the final exposure pattern. Figure 7 shows a comparison of patterns of e-beam exposure on LiNbO_3_ substrate with and without Au layer on resist. A schematic illustration also shows the effect of with and without Au layer on dissipating the charge during EBL exposures.

### 4.4. Correction of Proximity Effect

The proximity effect in EBL is the phenomenon that the electron scatters in the resist or the substrate at a significant distance from the incident beam cause the developed pattern to be wider than the scanned pattern, i.e., the proximity effect cause the electron beam resist outside the scanned pattern to receive a non-zero dose. The electron scatters could come from electron forward scattering and backscattering, which statistically broaden the e-beam in the resist.

Proximity effect will affect the size and shape of the exposure pattern obviously, which must be corrected for the requirement of a high precision pattern. There are three factors that affect the proximity effect, namely the energy of the incident electron beam, the substrate material, and the dose of the electron beam per unit area. In previous experimental analysis, the acceleration voltage and substrate material have been determined according to the design, thus the dose of the electron beam is only parameter that can be adjusted. Although the size and width of the design pattern can be adjusted by linear interpolation base on existing exposure data to make sure that the exposed patterns match the original design, the process is tedious and might be unavailable for some subtle or special exposure patterns.

**Figure 8:**
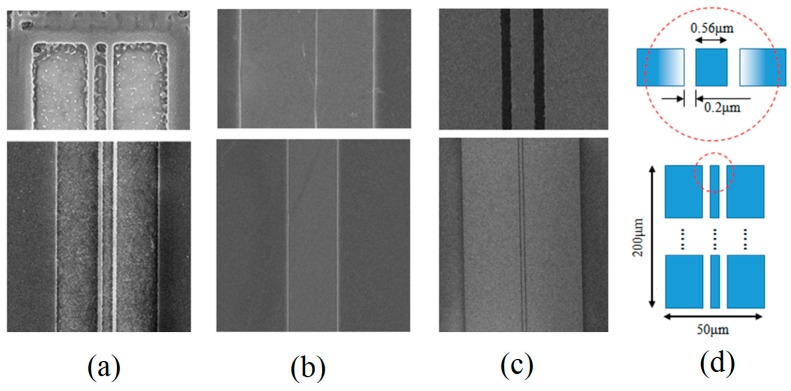
A comparison of developed patterns with an under-exposure (**a**), an over-exposure (**b**), and a modulation exposure (**c**) based on a specially designed modulation exposure graphics with the color intensity to display exposure does (**d**).

The modulation exposure is a good strategy, by which the exposure does can be adjusted and controlled according to the proximity correction algorithms for achieving uniform resist exposure. Figure 8 shows the influences of under-exposure, over-exposure, and modulation exposure on proximity effect. It can be seen from Figure 8a that the under-exposure due to low exposure will result in a large amount of residual resist that appear in the whole developed pattern. In contrast, if the exposure dose is suitable for the middle area, a proximity effect will result in an over-exposure in adjacent area where if a same exposure dose is still used, as shown in shown Figure 8b. In order to avoid above situation, the exposure dose used in the adjacent area can be adjusted in gradient change to correction the proximity effect. Figure 8c exhibits a perfect developed pattern when using a modulation exposure based on a specially designed modulation exposure graphics with the color intensity to display exposure does, as shown in Figure 8d. 

### 4.5. Characterization and Sensing Properties of SAW Devices 

**Figure 9:**
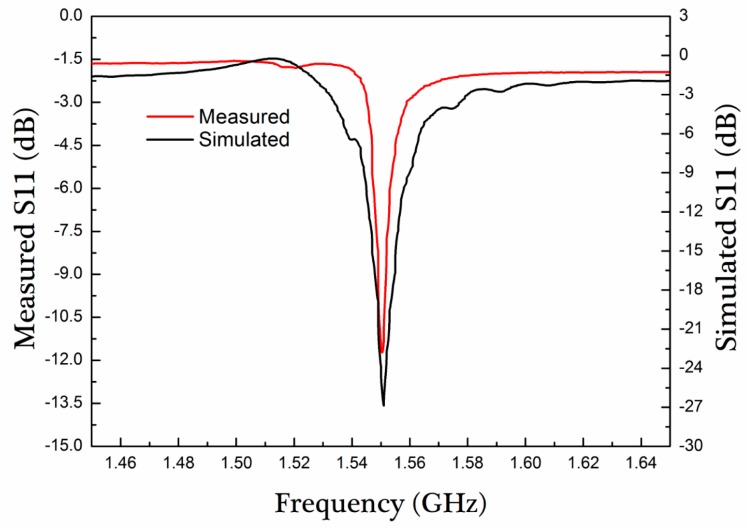
Response spectrum of S11 of SAW resonator based on 128° Y-X LiNbO_3._

Figure 9 shows a frequency responses comparison of the simulated and measured S11 of SAW resonator based on 128° Y-X LiNbO_3_. For the measured S11, the SAW resonator exhibits a Rayleigh wave resonance peaks at 1.550 GHz with return loss about −11.92 dB, and quality factor *Q* is 517 [30]. In contrast, for the simulated S11, the resonance peak is 1.532 GHz with return loss about −35.59 dB, and quality factor *Q* is 775. It can be seen that the frequency difference of both resonance peaks is about 5 MHz. At same time, there is a significant difference in return loss and Q, which is attributed in the impedance mismatching. The response spectrum of S11 was repeatedly measured several times in quite long period of time, and it was found that the resonant frequency remained fairly stable, which indicates that the SAW resonator can meet the needs of sensing applications.

**Figure 10:**
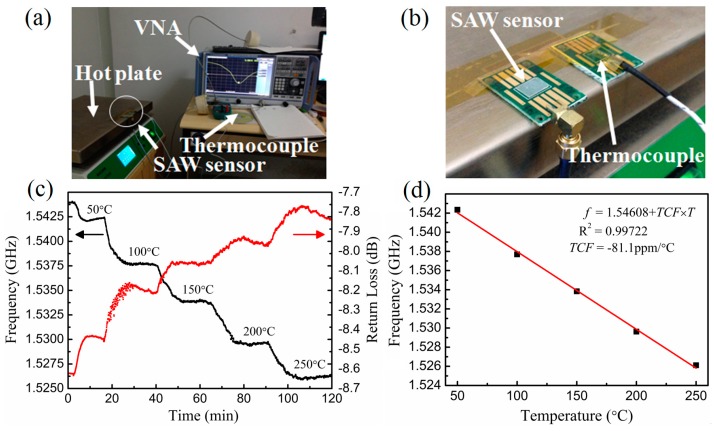
Photos of measurement setups (**a**) and LiNbO_3_-based SAW temperature sensor with a thermocouple to calibrate the temperature (**b**); F-t&R-t (**c**) and F-T (**d**) curves of SAW sensor.

The temperature sensing test of LiNbO_3_-based SAW resonator was performed in a hotplate with a PID controller, and the test data were recorded automatically by labVIEW software according to the test results of network analyzer (see Figure 10a). A thermocouple was used to calibrate the temperature of LiNbO_3_-based SAW temperature sensor (see Figure 10b). Figure 10c shows the frequency-time (F-t) and return loss-time (R-t) response curves of SAW sensor. It can be seen that the resonant frequency exhibit a step-down change when a step-up temperature was used from the range of 25 to 250 °C, and the total variation of return loss is round 1 dB in this range. Figure 10d shows the frequency-temperature (F-T) curves of SAW sensor. The resonant frequency is linearly proportional to the temperature with temperature coefficient of frequency (TCF) of 81.1 ppm/°C and temperature sensitivity of 125.4 kHz/°C.

**Figure 11:**
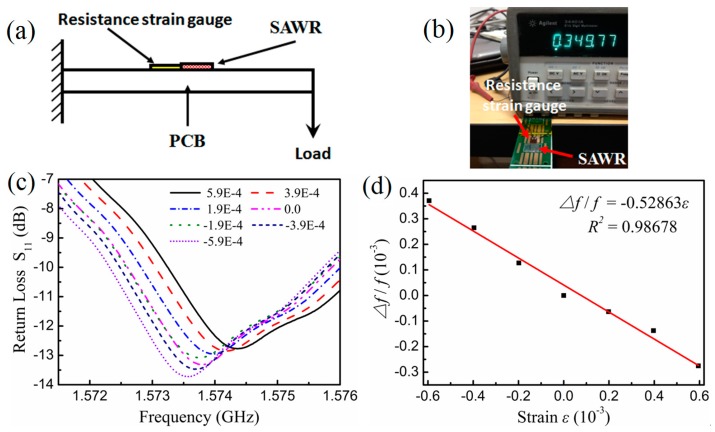
Schematic diagram of strain measurement method (**a**) and photo of LiNbO_3_-based SAW resonator attached to a PCB cantilever with a piezo-resistive strain gauge as calibration (**b**); S11 response of SAW resonator under different strains (**c**) and Δf/f - ε curves of SAW strain sensor (**d**).

Figure 11a shows a schematic diagram of strain measurement method of the SAW strain sensor. The strain sensing test of LiNbO_3_-based SAW resonator was performed in a PCB cantilever with a piezo-resistive strain gauge to calibrate the strain. As shown in Figure 11b, a SAW resonator and a 35 Ω piezo-resistive strain gauge with the sensitivity *K* of 2.0 and the strain limit of 2% were glued on the middle position of PCB cantilever. The strain *ε* can be calculated by the change in resistance of strain gauge, Δ*R* = *Kε*. Figure 11c shows the S_11_ response of SAW strain sensor under different strains. Experimentally, it was found that the positive strain (tensile stress) will down-shift the resonant frequency of SAW resonator while the negative strain (compressive stress) will up-shift the resonant frequency. Figure 11d exhibits a negative linear dependence of the frequency-shift change ratio Δ*f*/*f* on strain *ε*, with a strain coefficient of frequency-shift change ratio of −0.53 ppm/με and strain sensitivity of −831 Hz/με. This sensitivity can be further improved by using thinner LiNbO_3_ substrate and more sensitive package structure.

## 5. Conclusions

In conclusion, this paper presents the design, simulation, fabrication, and test of an *L*-band SAW resonator based on 128° Y-X LiNbO_3_ substrate. The design parameters of SAW resonator were optimized by the finite element (FEM) method and the coupling-of-mode (COM) theory. The SAW resonator with 600 nm linewidth was fabricated by the improved EBL technology. The use of double-layer electron beam resist and e-beam thermal evaporation were used to resolve the issue of incomplete lift-off of IDT structure. The issues of charge accumulation and proximity effect in EBL were eliminated by depositing a very thin layer of Au layer on the surface of the resist and the use of modulation exposure method, respectively. Experimentally, the LiNbO_3_-based SAW resonators fabricated using improved EBL technology exhibits a Rayleigh wave resonance peaks at 1.55 GHz with return loss about −12 dB, and quality factor *Q* is 517. Based on this SAW resonator, the temperature and strain sensing tests were performed, respectively. The experimental results exhibit a well linear dependence of temperature/strain on frequency-shift, with a temperature sensitivity of 125.4 kHz/°C and a strain sensitivity of −831 Hz/με, respectively.

## Figures and Tables

**Table 1:** Design parameters of SAW resonator. IDT: interdigital transducer.

Parameter	Symbol	Value
Bar-width of IDT	*W_i_*	600 nm
Bar-width of reflector	*W_r_*	600 nm
Period of IDT	*P_i_*	2.4 μm
Length of IDT aperture	*L_i_*	144 μm
Length of reflector	*L_r_*	144 μm
Pairs of IDT	*N_i_*	152
Bar number of reflector	*N_r_*	100
Distance between IDT and reflector	*d*	2.4 μm

**Table 2:** Coupling-of-mode (COM) parameters of SAW resonator (128° Y-X LiNbO_3_).

Parameter	Symbol	Value
Open circuit bar reflection coefficient (h/λ = 0.015)	*k_p_*	0.01958
SAW velocity (h/λ < 0.02, approximate free surface)	*V_sf_*	4032.4 m/s
SAW velocity (Open circuit reflection bar)	*v_og_*	3654.6.4 m/s
Static capacitance	*C_0_*	0.481 fF/μm
Propagation loss	*L_P_*	0.0035 dB/λ
Electrode square resistance (Au) (h_m_ is the electrode thick in um)	*r_s_*	0.034/h_m_·Ω
